# On the Creation of a Material Bond between L-PBF-Manufactured AZ91 and Ti-6Al-4V Components in the Context of Medical Applications

**DOI:** 10.3390/ma17184667

**Published:** 2024-09-23

**Authors:** Lennart Grüger, Felix Jensch, Fabian Dittrich, Sebastian Härtel

**Affiliations:** Department Hybride Manufacturing, BTU Cottbus Senftenberg, Konrad-Wachsmann-Allee 17, 03046 Cottbus, Germany; felix.jensch@b-tu.de (F.J.); fabian.dittrich@b-tu.de (F.D.); sebastian.haertel@b-tu.de (S.H.)

**Keywords:** AZ91, Ti-6Al-4V, additive manufacturing, L-PBF, implant technology

## Abstract

Within the scope of these investigations, the feasibility of a material bond between Ti-6Al-4V and the magnesium alloy AZ91 is analyzed. Ti-6Al-4V is frequently used for implants due to its biocompatibility, corrosion resistance, and specific strength. However, depending on the surface quality, the attachment behavior of the bone to the implant varies. Magnesium implants promote the regeneration of bone tissue and biodegrade as the bone tissue heals. Combining the properties of both materials in one implant enables a reduced implant volume and increased stability. For this reason, this study aims to demonstrate the feasibility of creating a material bond between the materials Ti-6Al-4V and AZ91. For this purpose, Ti-6Al-4V truncated cones and AZ91 sleeves were produced using the additive manufacturing process of laser powder bed fusion (L-PBF). The as-built sleeves were then pressed onto machined truncated cones. Since zinc serves as a lubricant and has good diffusion properties with the materials used as a result of heat treatment, a comparison was made between zinc-coated and the as-built Ti-6Al-4V samples. This showed that a bond was created after hot isostatic pressing and that the push-out force could be increased by more than 4.5 times. Consequently, a proof of feasibility was demonstrated, and a high potential for applications in medical technology was shown.

## 1. Introduction

### 1.1. Materials for Implant Applications

Currently, the various materials approved in medical technology are combined in joint replacements using different individual parts. The aim is to combine permanent and non-permanent or resorbable and non-resorbable materials [1]. However, a hybrid implant consisting of two different materials has yet to be created. Regarding the material combinations, the following applies: the greater the differences between the implant material and the bone in terms of mechanical properties, the greater the stress shielding. Stress shielding refers to the bone resorption caused by the inserted implant’s load shielding on the bone. This can cause a permanent implant to loosen over the years and results in the relative movement of the implant under load. Once the implant has loosened, the implant must be removed and replaced with a new one. This is a significant factor in the field of joint replacements [2].

**Figure 1:**
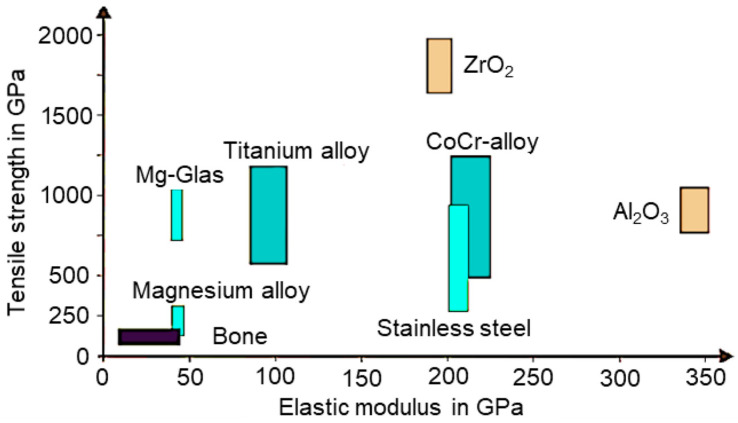
Overview of the material properties of metallic materials in medical technology compared to human bone according to [4].

Because magnesium has positive effects on bone growth, the combination of the materials magnesium and titanium within a hybrid implant is suitable. It leads to a significant improvement in usage properties, such as ingrowth behavior and longevity [3]. This is caused by the similar strength properties between magnesium alloys and human bone; see Figure 1 [4].

**Figure 2:**
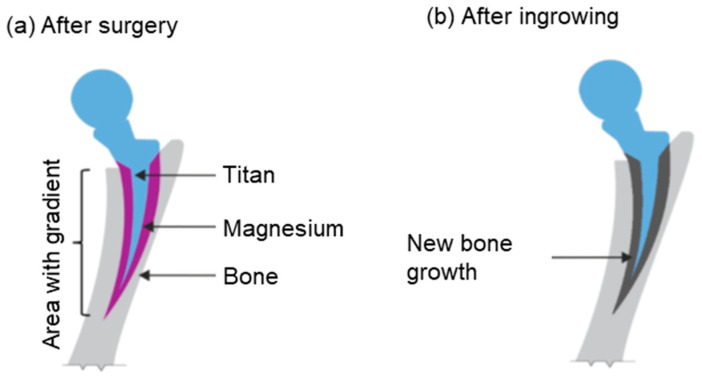
Illustration of the possible implementation of a hybrid implant.

Consequently, magnesium alloys are highly suitable compared to other resorbable material alternatives. The aim when creating hybrid implants is to match the resorption of the material as closely as possible to the growth rate of the bone and consequently optimize bone ingrowth. Titanium represents the gold standard of permanent materials and is used as the permanent base material in this study. Figure 2 shows an abstraction of the hybrid implant.

### 1.2. Titanium and Magnesium in L-PBF Processes

The laser powder bed fusion (L-PBF) process holds great potential for lightweight construction. As a result, research into titanium and magnesium materials is being pushed forward. Due to its good mechanical properties, corrosion resistance, and biocompatibility, the material Ti-6Al-V4 is approved for implants in Germany. Consequently, it is frequently used for additive-manufactured implants, as can be seen in different publications about cranial prostheses, auxiliary surgical components, shoulder blade prostheses, knee prostheses, dental implants, elements of the spinal column, acetabular cup implants, or hip and femur implants [5,6,7,8,9].

To ensure that the body integrates the metallic implant without problems, the inserted component must match the replaced bone and its structure as closely as possible. To achieve a suitable combination of elasticity and strength for the implants, Falkowska et al., Ataee et al., and Bartolomeu et al. examined various grid structures made of Ti-6Al-4V regarding their mechanical properties [6,7,8].

Another option for harmonizing the stiffness of bone and the implant is the targeted production of components with an increased porosity by varying the process parameters of laser power, exposure speed, and hatching distance [9]. The surface quality of the component plays a particularly decisive role in the connection of the implant to the body. The work of Alipal et al. [10] provides an overview of the surface requirements for additively manufactured implants. Since the surface erosion of the implant can lead to dangerous reactions with the organs, the material’s corrosion resistance is also very important. Based on the work of Sharma et al., it was shown that Ti-6Al-4V exhibits very high corrosion resistance in environments containing NaOH, SBF, and NaCl [11].

Magnesium alloys are suitable as the second component of the hybrid implant. These are also biodegradable, meaning they naturally degrade in the body and do not have to be surgically removed after healing. Their mechanical properties, similar to those of human bone, also enhance compatibility. Various studies have already recorded possible process parameters of magnesium, such as those by Wu et al. and Savalani et al. Ng et al. have investigated the process parameters and the resulting density and corrosion resistance of the manufactured components [12,13,14]. In addition, promising investigations with lattice structures for magnesium alloys were carried out, to improve the further adaptation of the mechanical properties between implant and bone and consequently improve biodegradability [15]. Matena et al. have demonstrated the promising properties of coated magnesium implants in a comparative study. The results are comparable with the already common titanium implants [16]. Vignesh et al., Kaushik et al., and Manakari et al. provide an overview of the current applications, challenges, and opportunities of additively manufactured magnesium components in biomedical engineering [17,18,19]. The studies clearly show that depending on the application and powder size, different parameter settings can lead to the desired manufacturing result.

### 1.3. Material Composites Made of Titanium and Magnesium

Both titanium and magnesium have a hexagonal structure at room temperature and are lightweight materials due to their low density. However, combining the two elements poses a major challenge. In particular, the large difference between the melting temperatures and the mutual solubility is the reason for this. The melting temperature of magnesium is approx. 649.5 °C and for titanium, approx. 1668 °C. In addition, a maximum of 1.6 at% magnesium is dissolved in titanium at 865 °C. At the same temperature, only 0.12 at% of titanium can be dissolved in magnesium [20]. This means there is a large mixing gap between the two elements, in which both elements solidify separately [20,21,22]. Consequently, direct solid-state diffusion is not possible. In addition to solid–liquid diffusion, which is difficult to implement, intermediate elements can be used for diffusion bonding in both directions, as shown in [23]. In this case, the boundary layer and the newly formed phases have the greatest influence on the mechanical properties of the diffusion bond.

The best-known alloy groups for magnesium are based on aluminum, manganese, and zinc, and for titanium alloys on aluminum, vanadium, tin, zirconium, niobium, vanadium, and molybdenum (see [22,24]). Due to their use in alloys, all these elements have already been examined for their suitability as intermediate elements. According to [25], the most promising elements are zinc, aluminum, and nickel.

For the manufacture of hip implants, the possibilities for creating the diffusion layer are limited by the geometry of the two components. Direct unidirectional heat input at the interface, as through friction or laser welding, is not possible. Accordingly, only solid-state diffusion up to the melting point of the magnesium alloy or molten diffusion through low-melting elements or eutectics can be used. However, solid-state diffusion requires a form closure. This cannot be completely guaranteed due to the component geometries. Consequently, transient liquid phase (TLP) bonding is an effective option. Zinc, aluminum, and nickel can be considered as interlayer elements. Using aluminum as the main alloying element of Mg- and Ti-alloys would be preferable. Using an additional aluminum layer leads to the formation of two eutectics at 450 °C and 437 °C on the magnesium side. However, the formation of many other intermetallic phases on the titanium side is problematic and difficult to control. Due to the high diffusion rate of aluminum in titanium, pores increasingly form on the aluminum side, as was shown in [26].

Zinc, with a melting temperature of 419.5 °C, and nickel, combined with the low-melting eutectic of MgNi at 508 °C, can also be used for TLP bonding. The two materials form further intermetallic phases until the solid solution is saturated. Compared to aluminum, these are mainly limited to TiNi_3_ and Mg_2_Ni or to MgZn_2_ for the zinc interlayer [27]. As a result of the eutectic reaction, the intermediate layer MgZn_2_ is formed almost exclusively. The reason for this is the acceleration of the diffusion process, which prevents the formation of intermetallic phases on the titanium side.

The intermediate layers are introduced into the diffusion system via coatings, pastes, or films. The layer thickness is a significant influencing parameter here. Due to the high corrosion tendency of magnesium, heat treatment in a vacuum or inert gas atmosphere is essential. Although the Mg-X-Ti composite is pressed at room temperature, a certain pressure must also be generated during diffusion annealing, especially when using the intermediate elements mentioned. This ensures that the shrinkage of the hybrid body does not lead to delamination of the boundary layer. Hot isostatic pressing (HIP) can be used to prevent deformation of the component geometry. In this process, atmospheric pressure is applied to the coated three-dimensional component surface. Tests on SUS 304 stainless steel have shown that the high pressure during HIP produces a defect-free diffusion zone and has a positive effect on the surface roughness [28].

This article aims to produce a material-locking connection between L-PBF-manufactured Ti-6Al-4V and AZ91 components utilizing suitable joining mechanisms. For this purpose, both uncoated and coated surface conditions will be considered. The final result should provide a basic understanding of the design and process chain for the production of a joint between titanium and magnesium alloys and thus serve as a starting point for further research into the production and application of such hybrid implants.

## 2. Materials and Methods

### 2.1. Materials

Powder of the magnesium alloy AZ91D, which was produced by Hana AMT Co., Ltd. (Cheongju-si, Republic of Korea), was used to manufacture the press-on sleeves. The powder has a chemical composition according to Table 1 and was processed with a particle size between 20 and 63 µm. The melting point of the material is 533 °C. The cones were made of the titanium alloy Ti-6Al-4V. The powder was produced by m4p materials solutions GmbH and also has a particle size of 20 to 63 µm. The chemical composition of the powder is shown in Table 1.

### 2.2. Methods

#### 2.2.1. Part Manufacturing

**Figure 3:**
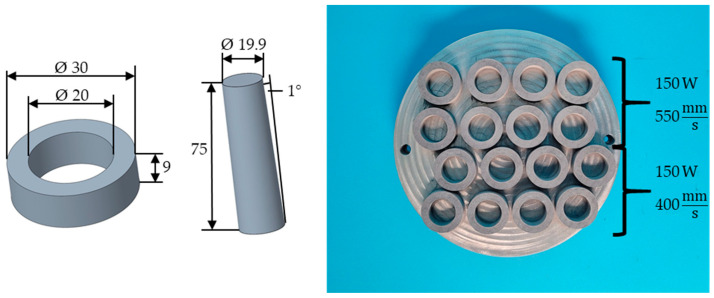
Geometry of the printed sleeves and cones (**left**), as well as the manufactured AZ91D sleeves, with the associated laser power and exposure speed (**right**).

The AZ91D sleeves were produced on an AconityMIDI (Aconity3D GmbH, Herzogenrath, Germany) equipped with a laser of 1070 nm wavelength with a focus diameter of 80 µm, under an argon atmosphere. A layer thickness of 30 µm and a hatching distance of 60 µm were used for all 16 components, whereby the substrate plate was not preheated. Before manufacturing the sleeves, suitable process parameters were determined in a previous test using a parameter matrix in which the laser power was varied between 150 W and 225 W in 25 W steps and the exposure speed between 400 mm/s and 850 mm/s in 150 mm/s steps. The two parameter sets of 150 W and 400 mm/s (sleeves 17 to 24) and 150 W and 550 mm/s (sleeves 25 to 32), which had the lowest porosity, were subsequently used to produce eight sleeves each. These were used to further realize and analyze a joint between Ti-6Al-4V and AZ91. Due to the use of the same manufacturing parameters, it can be assumed that sleeves 17 to 24 and sleeves 25 to 32 have approximately the same porosity of 0.77% and 0.44%, respectively, and surface quality (Figure 3).

The Ti-6Al-4V cones were produced on an EOS M290 (EOS GmbH, Krailling/München, Germany), whose laser also has a wavelength of 1070 nm and a focal diameter of 80 µm. The L-PBF process was carried out under an argon atmosphere, and the substrate plate was preheated to 35 °C. The various cones were produced using the same parameters: a laser power of 340 W, an exposure speed of 1250 mm/s, a hatching distance of 120 µm, and a layer thickness of 60 µm. The 75 mm long cone had a diameter of 19.9 mm on the narrow side, which increased at an angle of 1° to the wider side.

#### 2.2.2. Joining Components and Testing of the Joint

After production, the support structure was removed, and the sample height of the magnesium sleeves was standardized to 9 mm by machining. Furthermore, the inner diameters of the samples were measured. For the tests, samples with an inner diameter between 19.19 mm and 19.26 mm were selected. Accordingly, the geometric difference in the sample bodies in the thousandths range is considered to be negligible.

**Figure 4:**
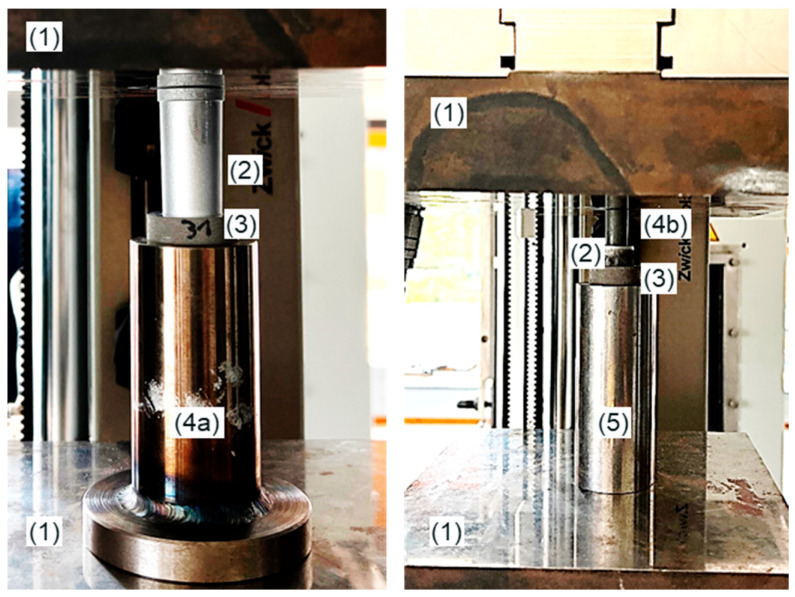
Visualization of the test setup for the press-on (**left**) and push-out test (**right**), (1) pressure plates, (2) titanium cone, (3) magnesium sleeve, (4a) counterholder press-on test, (4b) counterholder push-out test, (5) titanium cone holder.

The samples were pressed on and pushed out using the Zwick Z250 (ZwickRoell GmbH & Co. KG, Ulm, Germany) available at the Department of Hybrid Manufacturing. The associated test setup is shown in Figure 4.

For this purpose, the titanium cones were first machined to ensure a comparable surface quality and comparable geometries. Furthermore, the sleeve could be placed entirely on the conical surface. A pre-test was carried out to determine the maximum load capacity. In this test, a magnesium sleeve without a zinc intermediate layer was pressed onto the titanium cone until failure. The subsequent pressing of the sleeves followed the test plan shown in Table 2.

The first reference test (1) defined the reference force for the remaining tests. These were carried out under force control up to a press-on force of 1.72 kN. Force-controlled pressing was chosen to compensate for the possible influences of small geometry differences. As a result, the same press-on force results in a similar surface pressure for the samples. For a surface area of 551 mm², this results in a contact pressure of around 3.12 MPa. The zinc intermediate layer was applied with the zinc spray SF7800 Loctite (Henkel AG & Co. KGaA, Düsseldorf, Germany), which is heat-resistant up to 550 °C. This thermal resistance is important to avoid the vaporization of the zinc layer, which would inhibit the achievement of the targeted joint.

The samples with a zinc coating were then post-treated using the HIP process (Quintus Hot Isostatic Press QIH 15L (Quintus Technologies AB, Västerås, Sweden)). The process parameters were as follows:

- Temperature: 490 °C;
- Time: 3 h;
- Pressure: 100 MPa.

Following the HIP process, the push-out force of samples 2, 3, and 4 was determined. Specimen 5 was intentionally not tested in the push-out test. This was embedded for metallography and examined for a possible material bond using optical microscopy (VHX7000—Keyence Deutschland GmbH (Neu-Isenburg, Germany)) and scanning electron microscope (SEM) (Phenom XL Generation 2—FEI Deutschland GmbH (Dreieich, Germany)) examinations. Before the metallographic investigations, the sample was cut, embedded, ground, and polished (320 P Grit (Struers S.A.S, Champigny sur Marne, France)/9 µm diamond suspension (Struers S.A.S, Champigny sur Marne, France)/colloidal silica finishing polish (ATM Qness GmbH, Mammelzen, Germany)). The sample etching was unsuccessful, as the two metals react very differently to etching reagents (titanium needs strong reagents that would dissolve the magnesium).

## 3. Results

### 3.1. Results of the Press-On Process

**Figure 5:**
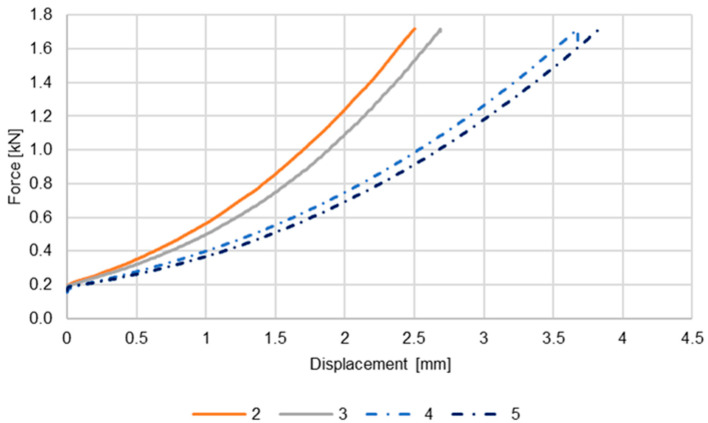
Force–displacement curve of the press-on process.

The press-on was carried out according to the test plan in Table 2. Figure 5 shows the force–displacement curves for these samples.

The curves for tests 2 and 3 show a similar increase. These reference tests were carried out identically and without the intermediate layer of zinc in order to calibrate the reference value. Accordingly, the only remaining influencing factor is the difference in the as-built structure of the inner diameter of the sleeve. This influence is considered negligible due to the slight difference in the curves and the inner diameter tolerance in the range of thousandths.

Tests 4 and 5 were carried out with an intermediate layer of zinc. These also show similar behavior, but the increase is less than with the dry pressed-on sleeves. The curves thus show that due to the lubricating effect of the coating, the friction between the sleeve and the cone could be reduced, resulting in the sleeve being pressed on over a longer distance.

### 3.2. Results of the Push-Out Tests

**Figure 6:**
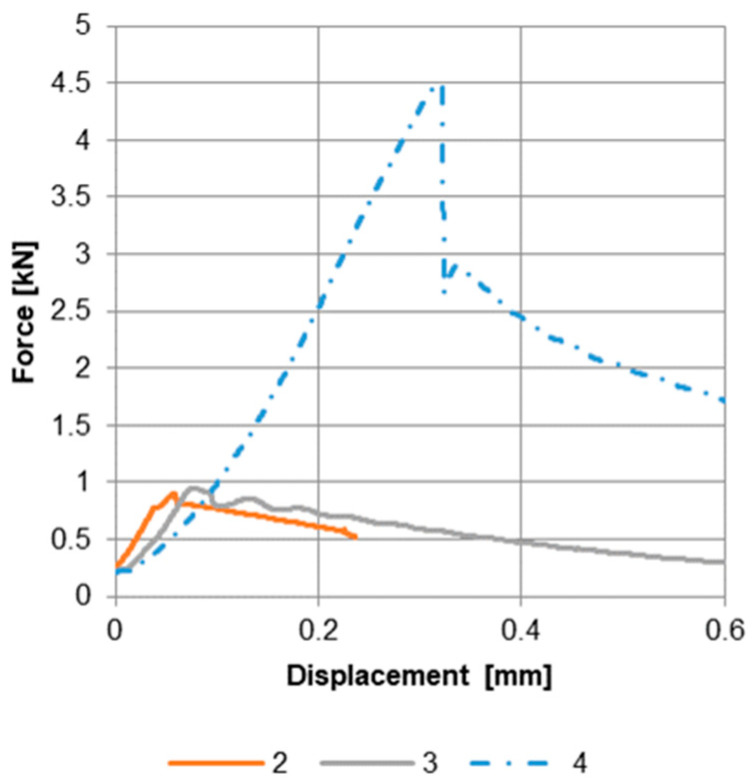
Force–displacement curve of the push-out tests.

The push-out tests were carried out following the press-on and the HIP process. The corresponding force–displacement curves and a comparison of the maximum push-out force are shown in Figure 6.

The analysis of the push-out force curves shows similar release forces for the dry-pressed samples. Sample 2 has a release force of around 0.9 kN, and sample 3 has a push-out force of around 0.95 kN. On average, this results in a push-out force of 0.93 kN. The push-out test of sample 4 is around 4.50 kN. Consequently, this is more than 4.5 times higher than the release force of the reference samples. Therefore, it is assumed that the applied zinc coating, in combination with the HIP process, has created a material connection between the titanium cone and the magnesium sleeve. This assumption is to be confirmed by the metallographic examination of sample 5. This sample was intentionally not subjected to the push-out test to perform metallographic analyses.

### 3.3. Results of Metallographic Investigations

**Figure 7:**
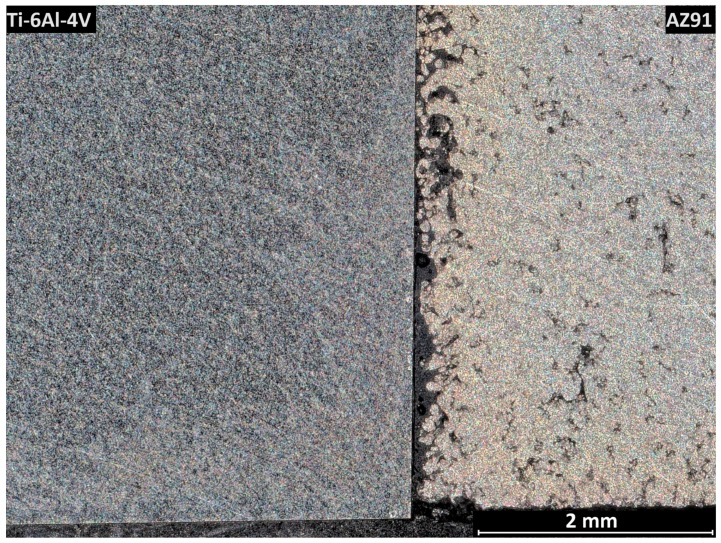
Overview of one side of the bonding interface of the Ti-6Al-4V cone (**left side**) and the AZ91 sleeve (**right side**). To avoid overexposure, the picture was taken in the dark-field mode of the VHX7000 microscope.

**Figure 8:**
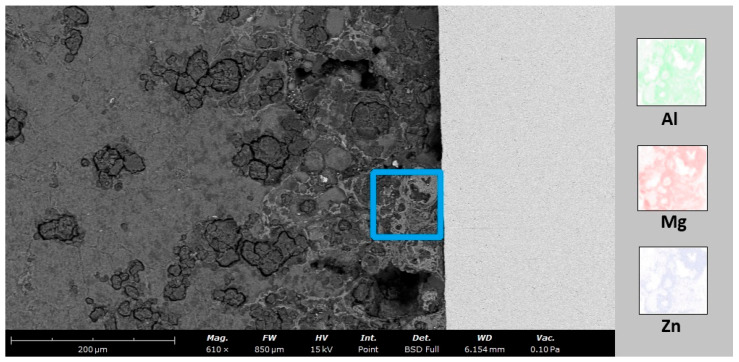
SEM image of the interface area with back-scattered electrons (**left side**, AZ91 sleeve; **right side**—light grey area, Ti-6Al-4V cone). The area of the EDS mapping is indicated with the blue rectangle. To the right, the element maps for Al, Mg, and Zn are presented, revealing a strong relation between the distribution of these elements.

The metallographic preparation of this material combination proved difficult. An overview of the bonding interface is shown in Figure 7. The dense Ti-6Al-4V is visible in the left part of the image, as well as the relatively porous AZ91 sleeve on the right side. The dark material visible on the interface is most likely the embedding material. The Zn bonding agent was not visible in the optical and SEM investigations. Due to its low melting point and solubility in magnesium and aluminum, it is assumed that upon melting, the zinc readily dissolves into the magnesium alloy, as supported by an EDS mapping shown in Figure 8. SEM investigations revealed multiple different features in the AZ91 sleeve at the interface to the Ti-6Al-4V cone.

## 4. Discussion

The bonding of titanium and magnesium presents a challenge, as mentioned at the beginning of this article. The reasons for this are the substantial differences in the melting temperature and the low diffusion rate of 1.6 atomic% magnesium in titanium and 0.12 atomic% titanium in magnesium [20]. Due to the resulting mixing gap, a bond in the solid state is immediately impossible [20,21,22]. Consequently, an interlayer of zinc was used as part of this work. This is considered one of the most promising intermediate elements [22,24,25]. However, the diffusion bond can only be created by applying pressure and temperature. Due to the high corrosion tendency of magnesium, the use of shielding gas is essential. The HIP process was used accordingly. This was applied at a temperature of 490 °C, corresponding to approx. 90% of the melting temperature of the magnesium; a pressure of 100 MPa; and a duration of 3 h. The comparison of the dissolution forces determined in the push-out tests shows that those of the heat-treated samples with a zinc interlayer are more than 4.5 times higher than the comparative values. Ohashi and Kaieda successfully achieved diffusion bonding at around 73% of the melting temperature of stainless steel [28]. In the test with the uncoated cone, a material bond could not be established. A bond between a zinc-coated Ti-6Al-4V cone and an AZ91 sleeve was achieved. However, the dynamics behind the bonding mechanism are not fully understood as yet. Multiple features in the AZ91 sleeve in the area of the Ti-6Al-4V interface are revealed by preliminary SEM investigations, leading to the assumption that the melting and dissolution of the Zn bonding agent in the AZ91 sleeve influences the phase formation and, ultimately, the bonding of both components. A more detailed analysis (SEM, XRD …) is necessary to understand the nature of the bond formed in these experiments. To improve the bonding of the components, a surface treatment for the inner surface of the sleeve should be considered, to close off any open porosity present at the surface. Generally, the porosity of the AZ91 sleeve could be considered beneficial, as it should promote tissue and bone growth when used as a medical implant.

## 5. Conclusions

Within the scope of the research, it was possible to create a bond between the titanium and magnesium materials. For this purpose, an intermediate layer of zinc was applied, and a HIP process was carried out at approx. 90% of the melting temperature of the AZ91 alloy. However, the initial investigations did not lead to a sufficient understanding of the bonding mechanics. Furthermore, the process parameters did not allow the full use of the as-built components. Accordingly, the Ti-6Al-4V truncated cone was reworked by machining. Nevertheless, it could be demonstrated that the resulting bond improves the push-out forces by more than 4.5 times compared to pure frictional adhesion. Consequently, there is great potential for applications in medical technology. Follow-up investigations aim to optimize the process parameters for the use of as-built structures and to investigate the influence of the surface roughness of the as-built structures, the zinc layer thickness, and the surface pressure.

## Figures and Tables

**Table 1:** Chemical composition [wt.%] of the used AZ91D and Ti-6Al-4V powder.

Material	Ti	Mg	Al	V	Fe	Zn	Mn
AZ91D	-	bal	8.00	-	0.009	0.97	0.2
Ti-6Al-4V	bal	-	6.33	3.98	0.16	-	-

**Table 2:** Visualization of the test design.

Test Number	Maximum Force [kN]	Traverse Distance [mm]	Zinc Intermediate Layer [Yes/No]
1 (Pre-Test)	to be determined	maximum	No
2	to be determined	2.5	No
3	1.72	to be determined	No
4	1.72	to be determined	Yes
5	1.72	to be determined	Yes

## Data Availability

The raw data supporting the conclusions of this article will be made available by the authors on request.
